# 

**Published:** 1896-02-15

**Authors:** 


### CONTENTS.

**Alcohol and Longevity** - 323

**The Influence of Mind upon Body** - 324

**Dyspepsia and Consumption.** By SOLOMON C. SMITH, M.D. 325

**The Ophthalmology of the Poets** - 326

**Annotations.**—Why do not Women Insure their Lives?—Who is to Judge?—Fifteen Reasons for Vaccination - 327

**The Institutional Workshop—**

DISCRETIONARY BEQUESTS - 335

PRACTICAL DEPARTMENTS.—The United States Army Medical Department - 336

**Notes and News** - 328

**Medical Progress and Hospital Clinics—**

The Treatment of Fractures of the Patella. By JOHN POLAND, F.R.C.S. - 329

PROGRESS IN SURGERY.—Abdominal Surgery - 331

Orthopædic Surgery - 332

THERAPEUTIC NOTES - 334

NEW APPLIANCES AND THINGS MEDICAL - 334

**Scraps and Gleanings** - 337

**The Book World of Medicine and Science** - 337

### EXTRA SUPPLEMENT.—THE NURSING MIRROR.

**News from the Nursing World.**—Incurable Cases at Westminster—Workhouse Infirmary Nursing Association—Nursing Guild at Richmond—Government Recognition of Good Nurses—Queen's Nurses at Tunbridge—A Generous Gift—Laundress or Nurse—The Midwives Bill—Irish Distressed Ladies' Fund—Wrexham District Nursing Society—The Company of Public Succour—Short Items - clxvii

**Lectures on Nursing.** X.—Food - clxix

**Nurses' Co-operation** - clxx

**St. John Ambulance Association Nursing Corps** - clxx

**Nursing in Canada** - clxx

**Chicago Woman's Club** - clxxi

**Nurses' Quarters at Bristol** - clxxi

**Our American Letter** - clxxi

**A Riviera Nursing Home** - clxxi

**Women Workers in New England Hospitals** - clxxii

**Doll Show and Competition** - clxxii

**On Certain Aspects of the Nursing Question as seen in England and Germany.** I.—Introductory - clxxiii

**The Book and Its Story** - clxxiv

**Everybody's Opinion** - clxxv

**Notes and Queries** - clxxv

**Where to Go** - clxxvi

**For Reading to the Sick** - clxxvi

